# Exit Music: The Experience of Music Therapy within Medical Assistance in Dying

**DOI:** 10.3390/healthcare8030331

**Published:** 2020-09-10

**Authors:** SarahRose Black, Lee Bartel, Gary Rodin

**Affiliations:** 1 Department of Supportive Care, Princess Margaret Cancer Centre, University Health Network, Toronto, ON M5G2M9, Canada; gary.rodin@uhn.ca; 2 Kensington Hospice, Kensington Health, Toronto, ON M5S2L1, Canada; 3 Faculty of Music, University of Toronto, Toronto, ON M5S2C5, Canada; lbartel@chass.utoronto.ca; 4 Department of Psychiatry, University of Toronto, Toronto, ON M5S1A8, Canada

**Keywords:** music therapy, assisted dying, end of life, MAiD, palliative care, phenomenology, qualitative

## Abstract

Since the 2015 Canadian legalization of medical assistance in dying (MAiD), many Canadian music therapists have become involved in the care of those requesting this procedure. This qualitative study, the first of its kind, examines the experience of music therapy within MAiD, exploring lived experience from three perspectives: the patient, their primary caregiver, and the music therapist/researcher. Overall thematic findings of a hermeneutic phenomenological analysis of ten MAiD cases demonstrate therapeutically beneficial outcomes in terms of quality of life, symptom management, and life review. Further research is merited to continue an exploration of the role of music therapy in the context of assisted dying.

## 1. Introduction

Within the scope of music therapy, certified music therapists often work with individuals who have life-limiting illnesses. Music therapy is formally defined by the Canadian Association of Music Therapists as a professional health discipline in which credentialed professionals use music purposefully within therapeutic relationships to support development, health, and well-being [1]. Music therapy has been practiced in hospice palliative care in a variety of modalities and intervention styles, including but not limited to clinical improvisation, during which client and therapist spontaneously create sounds on various instruments and/or with voice [2]; receptive music therapy, during which client listens to music that the therapist provides [3], also referred to as inter-active listening [4]; song-writing [5,6]; and active playing [7]. In providing care for those at end of life, music therapy goals may include pain and symptom management [8], emotional support through psychodynamic modalities [8], spiritual support through psycho-spiritual musical processes [9], strengthening of self-identity through intrapersonal exploration [10], and legacy work [10]. In palliative and end-of-life contexts, medical assistance in dying (MAiD) is now a legalized option in Canada, available to individuals who request the procedure if they meet the eligibility criteria [11]. 

There are a number of differences in the dying processes for those who receive MAiD and those who die of a terminal illness without medical intervention, such as having some control over the time of death rather than awaiting anticipated physiological changes with unknown timelines. Individual requests for music at the end of life may include choosing songs for various clinical experiences, interventions, or procedures. On these occasions, patients and families have requested live music for numerous reasons, although a primary motivation is most often to have a soothing, non-invasive distraction to providing relaxation and physiological ease [12]. Within the field of palliative care, music therapy is often used as an adjunct form of pain and symptom management and emotional support, and has become an increasingly utilized form of care in palliative and end-of-life settings [13,14]. Requests for music prior to and during MAiD have been noted to be a trend among those seeking MAiD [15]. Because music is used frequently at end of life in various contexts, ongoing research is needed regarding its benefits, potential usage, and outcomes. Within this context, further research is needed to understand how music therapy can best be utilized in order to establish best practices and improve its delivery. This qualitative phenomenological exploration of a music therapy experience within MAiD is the first of its kind. 

## 2. Background

Because assisted dying is a relatively new phenomenon in Canadian health care, a number of medical/allied health discipline organizations and professional associations have published clinical guidelines supporting their members in the navigation of MAiD [16,17]. In March of 2017, a group of individuals co-authored clinical practice guidelines intended to support Canadian music therapists in their own professional navigation of MAiD. In 2020, the practice guidelines were revised and republished through the guidance of a special interest group comprised of multiple healthcare professionals [18]. As the prevalence of assisted dying increases, various health care professionals have noted a marked increase in their involvement in the clinical process of assisted dying [19]. Due to the absence of research in the field of music therapy in this field, this qualitative study was designed to explore the role of music therapy within the context of MAiD from the perspective of patients, caregivers, and the music therapist involved, in order to begin to understand and further contribute to the clinical availability of interventions for individuals who choose MAiD. 

## 3. Methodology and Study Design 

This study sought to answer the following question: what is the experience of music therapy within the context of medically assisted dying for the patients, caregivers, and the music therapist involved? This question was posed within the methodological context of an overarching framework of interpretive-descriptive hermeneutic phenomenology, informed primarily by van Manen’s phenomenological approach [20]. This approach focuses on the act of observing and reflecting on lived experiences through discursive language and intentionally sensitive interpretive devices, allowing for analysis and thorough description [21]. 

The study design consisted of one to five individual music therapy sessions provided to all patient participants (depending on their requests, needs, and the availability of the therapist), followed by an interview with each caregiver participant (approximately three to four months after the patient’s death); the music therapist providing clinical care also served as researcher, and engaged in reflective phenomenological writing [20] and extensive field notes after each music therapy session and caregiver interview. The music therapist/researcher is specifically trained in qualitative methodology as well as self-reflexive practice. All music therapy sessions and interviews were audio-recorded and transcribed (no computer-assisted programming was used for transcription or analysis). Each component of the data underwent a hermeneutic thematic analysis, in addition to the primary researcher’s phenomenological descriptive, reflective writing during each case. Each participant’s experience was described through “phenomenological writing” [20]. Two researchers coded the data and an additional two researchers reviewed the findings, from which multiple themes emerged. 

This study was approved by the University Health Network’s Research Ethics Board (REB) (ID 18-5171, September 2018 through August 2019) and subsequently by the University of Toronto’s Research Ethics Board. 

### 3.1. Study Sites and Participant Eligibility

The study took place in a multi-site capacity between several hospitals and a residential hospice. The research team sought to recruit eligible patients (through a chart review and referral from the MAiD teams at each study site), as well an affiliated caregiver for each recruited patient. Caregivers (generally defined as a primary caregiver or companion) were identified by the patient participant. Patients were considered eligible to participate if they met the following criteria: (i) inpatients or outpatients who requested medical assistance in dying (MAiD) through their clinical teams; (ii) had a grievous and irremediable medical condition, whose deaths were reasonably foreseeable; (iii) age 18 or older; (iv) fluency in English; and (v) no cognitive impairment (as determined by their primary physician). Patients were considered ineligible if they had evidence of a cognitive impairment as assessed by their primary health care team. Caregivers were considered eligible to participate if they met the following criteria: (i) persons caring for a patient, (ii) age ≥18 years, (iii) fluency in English, and (iv) no cognitive impairment. Caregivers were excluded from participating and from being approached if they were known to have major communication difficulties as assessed by the researcher, or if the patient declined having a caregiver participant as part of the study.

### 3.2. Recruitment and Sample Size

The qualitative sample size aim was ten to twelve patient participants, eight to ten caregiver participants, and one music therapist/researcher. Through convenience sampling, patients were approached by a member of their primary health care team (e.g., physician, nurse, or allied health professional, in person at the hospital/institution or via telephone). The study was described, and the patient was asked if they were willing to be approached by a member of the study team; if they consented, a study team member approached and described the study in detail, outlining risks/benefits, and who to contact if further support would be needed. If the patient consented, the therapist/researcher met with the patient and planned/conducted music therapy sessions. No compensation was provided for participation. Recruitment ceased once saturation of themes was achieved. The total number of participants was 18, plus the addition of the therapist. All sessions were audio recorded and transcribed by either the researcher or the research assistant. Thematic outcomes were validated and triangulated by the study team. 

### 3.3. Participant Information 

Of 15 potential participants who were approached to be involved in this study, ten patients and seven caregivers completed the study. Table 1 outlines demographic information. All participants had metastatic disease progression of their initial cancer diagnosis. Two had comorbidities that contributed to their terminal illness: Participant #7 was in organ failure (lung) following a double lung transplant 13 years prior, in addition to a diagnosis of metastatic lung cancer. Participant #8 was living with HIV/AIDS in addition to metastatic adenocarcinoma. Of the three participants who died naturally of disease progression, two lost capacity to consent to MAiD due to functional, cognitive, and medical decline, and therefore were not able to go through with the MAiD procedure. One participant changed her mind about MAiD when she was transferred to a residential hospice and died naturally. Participant #1 received music therapy sessions beginning one month prior to her assisted death. Participant #2 received music therapy beginning one day prior to his assisted death. Participant #3 received music therapy sessions beginning two weeks prior to a natural death (though she was planning MAiD). Participant #4 received music therapy beginning three days prior to his natural death (though he was also planning MAiD). Participant #5 received music therapy beginning two months prior to her natural death (she changed her mind regarding pursuing MAiD). Participant #6 received music therapy beginning one week prior to his assisted death. Participant #7 received music therapy beginning one week prior to his assisted death. Participant #8 received music therapy two weeks prior to his assisted death. Participant #9 received music therapy 1 week prior to her assisted death. Participant #10 received music therapy exclusively on the day of her assisted death. 

### 3.4. Clinical Goals and Music Therapy Processes

Together with the music therapist/researcher, each participant created an initial clinical goal based on conversations around the role of music therapy, and an assessment by the music therapist/researcher. Instruments used in sessions included a keyboard, several pitched singing bowls, an ocean dream, and vocalizations/singing. Music therapy interventions included receptive (inter-active) listening, song-writing, psychotherapeutic processing through lyric analysis, and active playing/singing. Appendix A includes a list of some of the songs used in sessions. Table 1 includes a list of initial clinical goals, which functioned as a starting point for the therapeutic process. “Reminiscence” refers to an opportunity (initiated by the patient participant) to reflect on their life, significant life events, and any other reflections that felt pertinent or necessary with regards to their personal experiences. Music provided an associative link to various memories and experiences. “Comfort” refers to choosing music that provides a sense of being comforted by familiar associations that are linked to positive feelings, as well as a sense of ease and calm while listening to live music. “Symptom management” refers to use of music for pain control (distraction/diversion), and intentional breath management through guided breathing techniques. “Song-writing and legacy” refers to patient-created/therapist-supported songs that provide a therapeutic outlet for the patient’s creative self-expression and narrative creation, often gifted to a family member after the patient’s death.

## 4. The Experience of Music Therapy and Medical Assistance in Dying: Results

As a result of hermeneutic phenomenological analyses, cross-case thematic findings for patients were as follows (as outlined and detailed in Table 2): life reflection, control, communication and connectedness, and an aesthetic experience. With regard to life reflection, patients spontaneously engaged in retrospective reflection of previous life events, which they shared verbally and/or non-verbally (e.g., through music) with the therapist (and sometimes with their caregivers). Throughout many of the patient experiences, the therapist’s offer of music prompted patient participants to share life experiences, often connected to relationships, careers, rites of passage, and significant life events. At times, music told stories (e.g., through lyrics) and at other times, music served as a trigger or a prompt for the patient participant to tell their stories. For example, Participant #8 requested “I Will Remember You” by Sarah McLachlan because he felt that it “told his story”, in that it articulated how he felt about losing many friends to HIV/AIDS during the 1980s and 1990s, and it helped him articulate his feelings around the ending of his own life.

Control was an emergent theme, as patient participants were generally very particular about their musical choices as well as the sequence of events and rituals they planned for their MAiD intervention. Patient participants often spoke of wanting control over their end-of-life experiences and named music as an opportunity to control both the processing of emotion related to the experience and the specific events that took place during MAiD. For example, Participant #8 chose music that represented aspects of his life and shared that certain lyrics spoke to him because they represented part of his identity. Participant #1 chose music that reflected her experiences as a young woman in a refugee camp, and as an immigrant in a new country, which she spoke about at length in her music therapy sessions with her daughter. She shared that it was “important to tell these stories and share them while still able to”.

Music therapy sessions functioned as an opportunity for patient participants to communicate and connect with both themselves (intrapersonal) and their loved ones and clinical care team (interpersonal). This involved music as a means of sending a message to a loved one or connecting to oneself through associations that the music brought up in the patient participant. Participant #9 chose music that connected her directly to a personal experience of her faith; Participant #10 chose a song to sing before and during her MAiD intervention that was significant in her personal and professional life, and allowed her to create an opportunity in which she could connect with her loved ones by inviting them to sing with her during her assisted death.

The fourth emergent theme, aesthetics (musical pleasure as a catalyst for therapeutic outcomes) was notable in that patient participants found the aesthetic musical pleasure they experienced to function as a catalyst for therapeutic outcomes such as symptom management support, memory triggers, emotional processing, and identity exploration. Patients commented directly on the “beauty” of the music, noting that they found it “calming, beautiful and very soothing” (Participant #7), or “beautiful, so blissful” (Participant #4). The aesthetic experience of music appeared to offer visceral and physical sensations for patient participants, as well as an opportunity for ease and enjoyment.

Cross-case thematic findings for caregivers were as follows: immediacy of emotion, reflection, witnessing, and unexpected opportunities. Table 3 outlines primary and secondary themes.

Caregivers noted that music brought up a lot of emotion during the sessions, which often manifested as tearfulness, verbal reflections in conversation, and verbal sharing of experiences. Many caregivers commented that the music elicited emotion in a surprisingly immediate way, as they would often begin to cry shortly after the music began. Through follow-up interviews, caregivers shared that the music therapy created opportunities for their own reflections on personal narratives, including past experiences such as pivotal life moments with their loved one or current experiences of anticipatory grief. The wife of Participant #4 shared that she reflected on their marriage and life together while listening to the songs within the music therapy session, many of which played an important role throughout their relationship.

All caregivers indicated that they felt the music therapy played an important role in supporting their own witnessing of their loved ones’ emotional and narrative expressions. They noted that the music therapy sessions felt like a space in which they were bearing witness to their loved one’s journey, particularly the narratives that the patient was sharing (e.g., Participant #1 spoke at length about her childhood in Vietnam, while her caregiver daughter videotaped her stories and asked questions about her experiences).

The fourth emergent theme for caregivers was a sense of unexpected opportunities for life review through music. Caregivers consistently articulated their surprise in experiencing music therapy as an opportunity for their loved one to explore life events and significant moments. The caregiver (husband) of Participant #5 said that he was not expecting to receive a song that the participant wrote, but felt it was a “profound opportunity to reflect on her life in a positive way”. The caregiver (best friend) of Participant #6 mentioned that she was surprised by having the option to participate in music therapy sessions with her loved one at the hospice, and noted that it was a “welcome surprise and a chance for everyone to be together and reflect within the music”.

Cross-case thematic findings for the therapist/researcher emerged as trust, witnessing, therapeutic relationship immediacy, and navigation. Findings are further outlined in Table 4. Trust was noted to be developing between the patient and the therapist through the use of music. While therapist reflections indicated an ongoing sense of uncertainty (primarily in terms of the timing of MAiD and role of music therapy), the therapist’s reflections revealed a sense of feeling trusted by the patients and caregivers and trusting the music therapy process itself. One reflection noted, “I had to trust. I was nervous to go into the room…but I allowed myself to lean into it, be honest, and brave. And I felt like (Participant #8) was honest and brave too.” Witnessing the unfolding of narratives through music was a prominent topic of reflection within the therapist/researcher’s writing; being invited into a patient’s room, experience, and ultimately their death was a concept that surfaced repeatedly. The therapist/research writes, in reflection on Participant #9: “There is always so much under the surface. We are invited to witness the tip of the iceberg, if we are lucky, and that’s where I find myself. Witnessing.” In response to Participant #10: “I witnessed each patient and caregiver participant experience assisted dying in their own unique ways, with their own unique musical choices.” Therapeutic relationship immediacy was notable in that patients and caregivers were consistently sharing the intimate details of their lives, including emotional states, fears, challenges, concerns, and thoughts on death. Based on reflections, there was a deep level of intimacy and connection, as noted by patients’ tearfulness and their gesture of reaching out to hold the therapist’s hand, both often within the first session. The fourth prevalent theme was a sense of navigation, specifically of processes related to MAiD, in tandem with patients and caregivers. Patients and caregivers alike would ask the therapist/researcher for thoughts, advice, support, and reassurance around the steps of the MAiD application process. Because the experience of requesting and receiving MAiD has so many layers and components, it appeared that part of the music therapist’s role was to provide a safe space for patients and caregivers to share the complexities of their experiences.

## 5. Discussion

Each participant told their stories and shared their experiences with some variation, which parallels the notion that human beings favor highly unique musical choices, preferences, and needs, which depend heavily on pre-existing associations, current contexts, and personal experiences. The unique and personal relationship that each human being has to music may merit the presence of a music therapist who can personalize therapeutic interventions for each individual. Although each participant had unique needs, the trends in the themes were notable. The trends observed in patient participant data were often supported in caregiver interview data, and likewise in the researcher data. Though each overarching theme had slight variations between participants, there was an overall sense that music therapy was a positive and supportive intervention during the MAiD process. The findings of this research may support the development of music therapy practices in the context of MAiD. Because music therapy can offer a non-verbal approach to care and expression of emotion, music therapy may function as an opportunity for patients with communication difficulties (e.g., if they are unable to communicate verbally or if the patient and their healthcare providers do not speak the same first language). The positive associations that patients and caregivers had with music therapy and MAiD may provide incentives for institutions and all levels of government to increase funding for the arts in health care. Furthermore, these initial results offer the groundwork for further research in this previously unexplored area, leading to potentially optimizing patient care by offering an evidence-informed and personalized intervention for support prior to an assisted death. As MAiD becomes increasingly available, and as legislation continues to shift, these findings may serve as an incentive to further explore the application of music therapy during MAiD in a variety of populations and settings, for example, within home care or long-term care settings. The addition of these findings to the broader music therapy and end-of-life discourse may have applications in educational settings, particularly in the training of medical students, music therapy trainees, and other allied health care professionals who may work with those requesting MAiD.

## 6. Limitations

The relatively small sample size was only able to capture a fraction of the data that may have come of a study that recruited for much longer with a wider scope and with additional therapists. The dual role played by the therapist/researcher has limitations, in that there is a natural bias in having the same person take on both roles. However, the therapist/researcher’s own front-line experiences allowed for a window into the world of music therapy and MAiD that would be inaccessible to a secondary researcher. This position allowed the therapist/researcher to be intimately connected to the experiences of the participants. The steps to both acknowledge the bias and to triangulate the data analysis included the support of a research assistant (TK), whose roles included transcribing many audio-recorded sessions, as well as conducting a subsequent analysis of the data, which was then compared, contrasted, and integrated into the results. Data was also shared with the co-supervisory team for their critical feedback and expertise. Additionally, the questions that were used as prompts within the music therapy sessions as well as the caregiver interviews were reviewed by a number of qualitative research experts. The questions did not enquire about the relationship between the therapist and the participants; however, themes emerged through other means, although not specifically prompted.

## 7. Conclusions and Future Directions

While this initial study provided groundwork for entering into the lifeworld of patient, caregiver, and therapist participants, much is left to learn. An exploration of more music therapists’ experiences would enrich the sole music therapist perspective provided in this study. Further research objectives may include a deeper exploration of patients’ perceptions and experiences, and if or how music therapy might affect physical symptoms prior to assisted death. Symptom experiences were not specifically examined, and may add to the data set via a quantitative approach in a future study. If music therapy was offered earlier in some patients’ care trajectories, physical, emotional, and psychosocial outcomes may have varied. Another direction for future research might involve evaluating the benefit of music therapy support groups for bereaved family members of patients who had an assisted death, perhaps in comparison or contrast to bereaved family members of patients who had a natural death. In order to understand more deeply the impact of music therapy of music therapy in the context of medical assistance in dying, future research is merited and necessary.

## Figures and Tables

**Table 1:** An overview of patient participant demographics.

#	Age	Diagnosis	Total # Sessions	Death Type	Death Location	Caregiver	Initial Clinical Goal
1	57	Metastatic ovarian cancer	2	MAiD	Home	Daughter	Reminiscence
2	69	Metastatic prostate cancer	1	MAiD	Cancer centre	Daughter	Comfort
3	60	Metastatic lung cancer	5	Natural	Cancer centre	N/A	Symptom management
4	66	Metastatic lung cancer	1	Natural	Cancer centre	Wife	Symptom management
5	60	Metastatic ovarian cancer	3	Natural	Hospice	Partner	Song-writing/Legacy
6	67	Met pancreatic cancer	2	MAiD	Hospice	Friend	Symptom management
7	69	Metastatic lung cancer	3	MAiD	General hospital	Daughter	Reminiscence
8	53	Metastatic anal cancer	2	MAiD	General hospital	N/A	Reminiscence
9	86	Metastatic lung cancer	1	MAiD	Hospice	Daughter	Reminiscence
10	75	Metastatic ovarian cancer	1	MAiD	Hospice	Husband	Music during MAiD

**Table 2:** Primary and secondary themes for patients.

Themes	Primary	Secondary	Secondary	Secondary
THEME 1	Life reflection (through the immediacy of musical interaction, music as narrator and as trigger)	In a time of moving towards death.	As witnessed by and engaged in with caregiver and/or therapist.	As triggered by musical choices.
THEME 2	Control (over choices: musical, ritual, physical)	Death acceptance as a form of control.	Control of musical choices for the purpose of personal identity representation.	Control over choice of ritual and sequence of events.
THEME 3	Communication and connectedness (with the self and others through music)	Music as an invitation to connected and communicate (between loved ones, between patients and loved ones, and between patients and clinicians).	Music as an act of exploring and understanding relationships.	Expression of emotion.
THEME 4	Aesthetic pleasure (musical pleasure as a catalyst for therapeutic outcomes)	Music as a catalyst for memories and identity representation.	Music as a visceral, physical, and associative experience of pleasure.	Aesthetic properties of music as a source of symptom management support.

**Table 3:** Primary and secondary themes for caregivers.

Themes	Primary	Secondary	Secondary
THEME 1	Immediacy of emotion (access to emotion through music)	Music creates a holding space that allows for immediate access to emotion.	Music connects to emotional content with immediacy.
THEME 2	Reflection (on personal narratives within the music)	Music invites a retrospective reflection into the caregiver’s experiences.	A contextual reflection period is created (within the music).
THEME 3	Witnessing (emotional and narrative expression)	Witnessing patient’s experiences through the lens of music.	Interconnectedness between patient narratives and caregiver narratives.
THEME 4	Unexpected opportunities (for life review through music)	Opportunity to engage in life review.	Opportunity to express unexplored/ unarticulated emotion.

**Table 4:** Primary and secondary themes for the therapist/researcher.

Themes	Primary	Secondary	Secondary
THEME 1	Trust (in the midst of uncertainty)	Acknowledging and empathizing with the uncertainty of the patient; many unknowns in the MAiD process.	Trusting the patient as navigator, and therapist points out possible routes within the music.
THEME 2	Witnessing (the unfolding of narratives through music)	Witnessing intimacy of relationships in the patients’ and caregivers’ lives.	Witnessing life and death narratives unfold through musical requests.
THEME 3	Therapeutic Relationship Immediacy (formation and development through music)	Creating and maintaining the holding environment.	Relationship development between patient and therapist expedited in the music.
THEME 4	Navigation (of MAiD processes, in tandem with patients, caregivers and the music)	Navigating timing of sessions within the context of MAiD.	Trusting the music as a co-therapist.

## Appendix A. An Abbreviated “Exit Music” Playlist

The following list includes some of the songs requested and utilized within patient participant music therapy sessions over the course of the data collection.

- The Rolling Stones: Wild Horses
  - Played and sung by music therapist during Participant #6′s MAiD procedure
- Leonard Cohen: Hallelujah
  - Played and sung by music therapist/researcher along with Participant #10 and her family during her MAiD procedure
- J. Pachelbel: Canon in D Major
  - Played by music therapist during a session with Participant #7 and used as an opportunity for reflection on significant life events; also played by music therapist during Participant #7′s MAiD procedure
- Stan Rogers: Northwest Passage
  - Played and sung by music therapist and Participant #4′s wife during a music therapy session as a vehicle for self-expression and reflection on Participant’s love of folk music
- Sarah McLachlan: I Will Remember You
  - Sung by music therapist during a session with Participant #8, upon request, as a tool for catharsis and processing upcoming death
